# YIM-P22. Extracellular vesicle induced macrophage differentiation; developing a disease in a dish model

**DOI:** 10.1186/1546-0096-12-S1-Y3

**Published:** 2014-09-17

**Authors:** Genoveva Keustermans, Oscar Van Den Bosch, Berent Prakken, Wilco de Jager

**Affiliations:** 1 LTI, Utrecht, Netherlands; 2 University Utrecht Medical Center, Utrecht, Netherlands; 3 Pediatrics, University Utrecht Medical Center, Utrecht, Netherlands

## Introduction

Extracellular vesicles (EVs) are small membrane vesicles of endocytic origin. EVs can be found in a plethora of body fluids and are known to carry both vesicle as well as parental cell specific proteins. The content of the particles is dependant on the stimulation and/or micro-environment surrounding the donor cell. Content analyses reveal the presence of proteins involved in targeting, adhesion, membrane trafficking, signal transduction, chaperones, enzymes and functional mRNA and miRNA, with particle release being a highly regulated process induced by biological, chemical or mechanical stimuli. EVs play a functional role in immune responses providing both activating and suppressive signals to target cells. This is exemplified by the fact vesicles from PMA-differentiated THP-1 cells have been shown to induce naïve THP-1 cell differentiation. Systemic juvenile idiopathic arthritis (sJIA) is a subtype of childhood arthritis that is characterized by its systemic manifestations, severe symptoms and, on a cellular level, polarization of monocytes to a pro- or anti-inflammatory phenotype depending on the active/remissive state of the patient.

## Objectives

EVs are biological vectors, carrying important messages from one cell to another. We aimed to develop a system in which the influence of sJIA patient plasma vesicles could be analyzed in order to determine their influence on monocyte differentiation and phenotype.

## Methods

THP1 cells where cultured and differentiated into macrophages. Upon differentiation into macrophages, cells where further skewed toward a pro– or anti– inflammatory phenotype. Vesicles where subsequently harvested from the culture supernatant of differentiated cells and placed on undifferentiated THP1 or M0 macrophages. Cytokine production and cell surface markers were used as read out for cell differentiation.

## Results

Upon polarization, elevated CD80 signified M1 differentiation while CCL22 was upregulated in M2-polarized macrophages. Vesicles harvested from M1-polarized macrophages skewed naïve macrophages towards an M1 phenotype while M2 vesicles may skew macrophages towards a more regulatory phenotype.

## Conclusion

Data provides evidence towards the functional role of EVs in macrophage differentiation. By optimizing our system, this model could be used to analyze the function and activity of EVs in sJIA, allowing us to determine the influence of these particles on disease pathology and possibly inferring the future active or remissive state of a patient.

## Disclosure of interest

None declared

